# Management of the Amniotic Band Syndrome with Cleft Palate: Literature Review and Report of a Case

**DOI:** 10.1155/2017/7620416

**Published:** 2017-01-26

**Authors:** Carolina Cortez-Ortega, José Arturo Garrocho-Rangel, Joselín Flores-Velázquez, Socorro Ruiz-Rodríguez, Miguel Ángel Noyola-Frías, Miguel Ángel Santos-Díaz, Amaury Pozos-Guillén

**Affiliations:** Pediatric Dentistry Postgraduate Program, Faculty of Dentistry, San Luis Potosi University, 78290 San Luis Potosí, SLP, Mexico

## Abstract

Amniotic Band Syndrome (ABS) is a group of congenital malformations that includes the majority of typical constriction rings and limb and digital amputations, together with major craniofacial, thoracic, and abdominal malformations. The syndrome is caused by early rupture of the amniotic sac. Some of the main oral manifestations include micrognathia, hyperdontia, and cleft lip with or without cleft palate, which is present in 14.6% of patients with this syndrome. The purpose of this report was to describe the clinical characteristics and the oral treatment provided to a 6-month-old male patient affected with ABS with cleft lip and palate.

## 1. Introduction

Amniotic Band Syndrome (ABS), also known as ADAM (acronym for* Amniotic Deformity*,* Adhesions,* and* Mutilations*), is a rare condition consisting of a broad group of congenital malformations caused mainly by the rupture of the amniotic sac, which produces a series of alterations due to the appearance of fibrous mesodermal amniotic tissue bands [1]. The syndrome exhibits different clinical manifestations at birth, such as constriction rings and limb and digital amputations, together with diverse craniofacial malformations and thoracic-abdominal wall anomalies [1, 2]. These defects represent disruptions not occurring along the known lines of embryologic development [2, 3].

According to recent epidemiologic data, the occurrence of ABS is around 1 in 1,200–15,000 live births and it exhibits no special preference for a specific gender or race [4]; however, some studies report a slight preference for Afro-Caribbean individuals [2]. The pathogenesis of the ABS has not been totally elucidated, but it probably has a genetic origin. Two theories have been proposed to explain the multiple causal factors associated with this syndrome. First, the* intrinsic model*, described by Streeter in 1930 [5], which suggests the existence of an early embryolesion with alterations of the germinal disc that would produce an inflammatory response of the adjacent amnions and that would then develop a fibrous band. Second, the* extrinsic model*, the more widely accepted theory, developed by Torpin in 1968 [6], in which the authors proposed that the rupture of the amnions during early pregnancy allows the embryo or fetus to enter into the chorionic cavity and to contact the chorionic side of the amnions. Thus, fetal structures may be trapped by the fibrous septum that protrudes into the chorionic cavity. Compression and adhesion of these amniotic bands, which float freely, may cause disruption of the fetal structures [7]. The fetus' arms and legs, tangled around the amniotic bands, may be amputated during intrauterine development due to loss of blood flow [4, 8, 9]. The variability in the type and severity of the anomalies caused by this syndrome can be attributed to the moment at which the amniotic membranes rupture. Other related manifestations comprising congenital heart defects, renal anomalies, polydactyly, supernumerary nipples, and skin tags [2].

In their reviews, Muraskas et al. [10] and Bouguila et al. [11] mention that the most common craniofacial anomalies characteristic of the syndrome include corneal and orbital defects, anencephaly, meningocele or encephalocele, palpebral colobomas, nose malformations, and facial nerve paralysis; in the oral cavity, there may be micrognathism, hyperdontia, and cleft lip with or without cleft palate, representing 14.6% of patients who are afflicted with this condition [12, 13]. Multiple anomalies are present in 77% of cases [10].

Prenatal diagnosis of ABS, as early as 12 weeks of gestation, is performed in 29–50% of cases, depending on the severity of the disorder and the time when the lesions appear [10, 14]. Diagnosis of the syndrome consists of the identification of a fibrous band that deforms the distal part of a body limb, which renders it difficult for the limb to move [15]. Fetal compromise may be suspected with the use of Doppler studies, which exhibit reversal of end-diastolic flow in the umbilical artery [10]. When the child is born, it is possible to confirm the diagnosis of ABS by performing a histopathological analysis of the placenta, which can inform about persistence of the amniotic chorionic rupture [16].

The purpose of this report was to provide a review of the literature related to the syndrome and to describe the case of a 6-month-old male patient with ABS, his systematic, craniofacial, and oral clinical characteristics and reconstructive/oral management provided.

## 2. Literature Search Strategy and Results

An exhaustive Web literature search of relevant references was conducted in March/June 2016 in the following five Internet databases, without language or publication-date restrictions: MEDLINE (via PubMed); EMBASE (Elsevier Science); Google Scholar; Latin Index; and Scielo. The study selection criteria were methodological designs comprised of clinical trials, cohort and case-control studies, and clinical case reports, carried out on infants, children, and adolescents. Articles had to include any type of relevant oral management process or intervention, such as diagnosis tests, craneofacial surgical/rehabilitation procedures, or dental restorative treatments; in vitro studies were excluded. The main search algorithm was the following: ((“Amniotic Band Syndrome” [Mesh]) OR (“Amniotic Deformity” [Mesh]) AND (“Pediatric Dentistry”) OR (“Maxillofacial Surgery” [Mesh])). Other search terms employed were as follows: “Craniofacial Anomalies”; “Orofacial Cleft”; “Lip Cleft”, and “Palate Cleft”, or “Pediatric Patients”, “Pediatrics”; “Children”; “Childhood”; “Child Dentistry”, and “Dentistry for Children”, all of these alone or with their different combinations. The filter “Age” was set at “Child: birth–18 years.” Subsequently, titles, abstracts, and keywords were objectively and independently reviewed. The papers selected were retrieved in full-text and read in detail. In addition, the authors hand-searched the content pages of the reference lists of these papers.

The literature search identified a total of 145 potential citation references; after reviewing titles and abstracts, 107 of these clearly did not meet the desired criteria and were discarded. The full-text of the remaining 38 citations was retrieved and screened in greater detail; finally, 28 relevant papers were identified for inclusion in the literature review, including 7 dental and 3 medical case reports.

## 3. Case Report

The patient was a 6-month-old boy who was born at 37 weeks of pregnancy who was referred from the Department of Maxillofacial Surgery of Hospital Central “Dr. Ignacio Morones Prieto” to the Pediatric Dentistry Postgraduate Clinic (San Luis Potosi University, Mexico) for a dental examination and the possible placement of a palatal obturator.

In relation to the perinatal medical history, the child's parents were healthy, nonconsanguineous at 39 and 38 years of age; the mother reported vaginal bleeding during the first month of pregnancy and a urinary tract infection in month 2 of pregnancy. At that time, the mother began prenatal care, including the intake of folic acid. As part of the medical history, no hereditary anomalies were reported, nor smoking, alcoholic-beverage consumption, or drug abuse during pregnancy. The baby-in-question was the mother's fourth pregnancy and he was born within the normal time period (body weight: 2,800 g, size: 51 cm).

**Figure 1:**
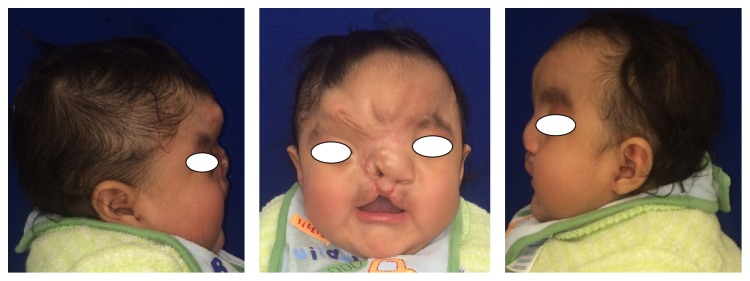
Extraoral views.

**Figure 2:**
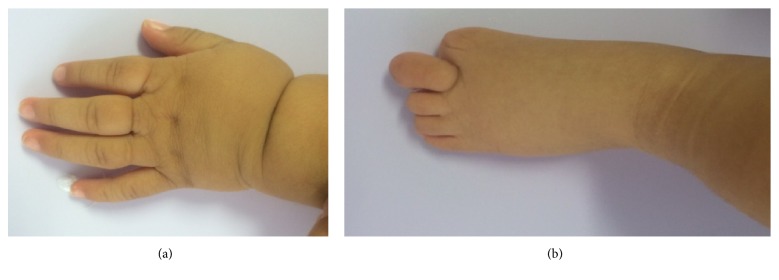
(a) Right hand. (b) Right foot.

**Figure 3:**
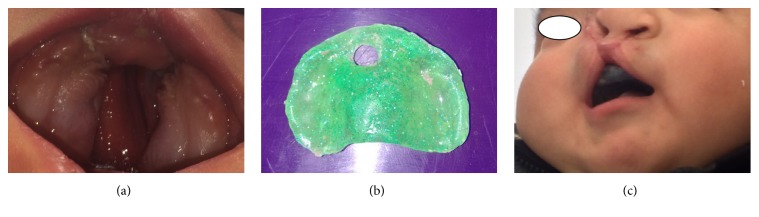
(a) Palate cleft view. (b) The obturator device (the hole was created to permit the recent eruption of the right central incisor). (c) Intraoral placement of the obturator.

Clinically, the patient exhibited multiple craniofacial anomalies, including a fissure on the upper part of the face, encephalocele, corneal opacity of the right eye, hypertelorism, severe hearing loss on the left side, and evident asymmetry (Figure 1). The remainder of his body manifested constriction rings and anomalies in hands and feet (amputation and lymphedema) (Figure 2). The intraoral clinical examination demonstrated a “Y”-shaped lip-palatal fissure from the soft palate terminating with a bifurcation in the upper lip and nose (Figure 3). A karyotype study was performed, whose result revealed the absence of chromosomal alterations. The final ABS diagnosis was determined based on the clinical findings. The patient underwent a reconstructive surgical procedure at 5 months of age.

Based on the intraoral clinical findings compiled, we decided to provide instruction to the parents with oral-hygiene indications, particularly on the cleaning of the infant's alveolar ridge with wet gauze at least once a day, preferably at night after the patient's finishing drinking his bottle. A condensation silicone impression was obtained of the upper dental arch for the fabrication of a palatal obturator; the device was fabricated using autopolymerizable acrylic. After placement of the obturator, its use was recommended for 24 hours a day, with a thoroughly washing each night. Then, control and review appointments were scheduled every 3 months in order to observe how well the palatal obturator adapted and to monitor the patient's craniofacial/oral development and the dental eruption process.

**Figure 4:**
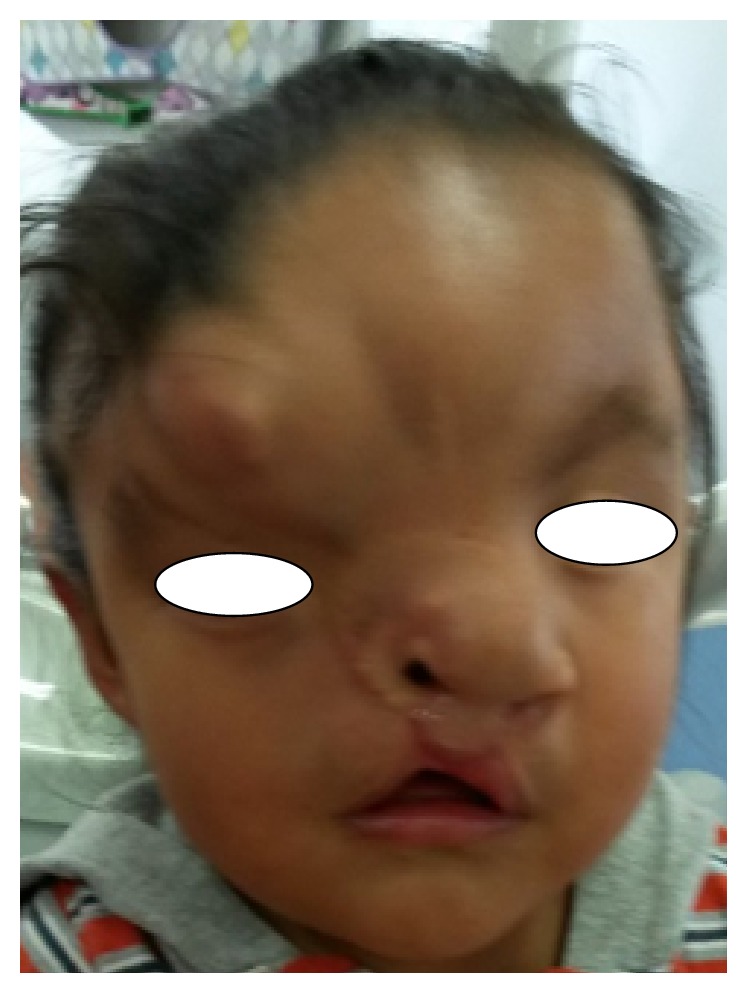
The patient's facial view, 10 months after the first examination.

**Figure 5:**
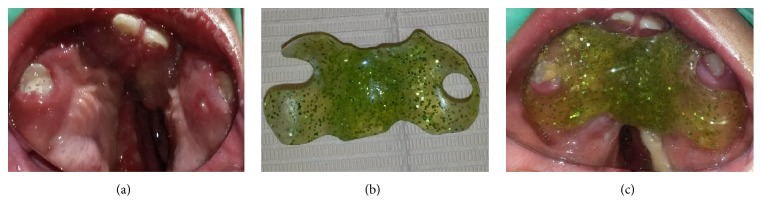
(a) Most recent view of the maxillary arch. (b) Adjustments made to the palatal obturator, according to the eruption process of primary teeth. (c) Palatal obturator well adapted in mouth.

The most recent oral/general examination appointment was 10 months after the first examination. According to the patient's mother, the child was in good general health. He showed a brachyfacial pattern and midface/maxillary arch hypoplasia (Figure 4) but a poor oral-hygiene level; the obturator had been properly used, and marked improvement in swallowing and feeding during the previous 3 months was manifested. However, the device was not well adapted because the upper primary central incisors and first molars were in the eruption process; therefore, the corresponding acrylic trimmings were performed (Figure 5). At the same time, we reinforced the hygiene instructions and began to apply topical high-concentration fluoride varnish on the enamel of these erupting teeth. The manufacture of a new palatal obturator was programmed in the following 4 weeks. Additionally, control and review appointments were scheduled in order to monitor the patient's craniofacial/oral development and his dental eruption progression. Thus, in the short/medium term, we will be able to plan the next reconstructive surgical procedure.

## 4. Discussion

The occurrence of the ABS with facial/lip/palate is a sporadic event; around 50 medical and dental cases have been reported in the literature over the past 25 years [4, 17]. Although this disorder includes diverse anomalies of the craniofacial and body structures, it is very difficult to specify exclusive clinical features [9, 18]. The observed variability in the type and severity of the anomalies depends on the time of rupture of the amniotic membranes; if this occurs during the first 45 days of pregnancy, the defects are more severe, mainly in the craniofacial structures, along with other anomalies of the central nervous system, visceral defects, and limb anomalies (such as syndactyly or polydactyly). However, if the fibrous bands appeared after week 12 of pregnancy, the characteristic adherences and constriction rings would only be evident at birth [12, 13, 19]. In the present case, the amniotic membranes' rupture probably took place during the first 45 days of pregnancy, according to the anomalies observed in the patient, such as encephalocele, facial fissure, constriction rings, and toe amputation [18, 20, 21].

Management of patients with ABS requires a multidisciplinary approach with a collaborative medical/dental team. Depending on its severity and the malformations present, the team is integrated by diverse specialists, such as Orthodontists, Pediatric Surgeons, Plastic and Maxillofacial Surgeons, Ophthalmologists, Neurologists, Geneticists, and Psychologists [22, 23]. In addition to being familiar with ABS, Pediatric Dentists must also collaborate actively, both in the prevention and the rehabilitation of oral-dental anomalies, bearing in mind the importance of active early intervention [1, 10]. Considering these issues, some authors have recommended a relatively novel procedure, the fetoscopy or in uterosurgery [10], with the intent of preventing limb amputation and repairing other small or complex ABS malformations. Basically, the procedure consists of releasing the constriction membranes of the limbs at risk; this therapeutic option is slightly invasive and is performed during the early stages of pregnancy [3], and its prognosis depends on the severity of the disorder [8, 23].

For the patient reported in the present work, the pediatric dental treatment provided consisted mainly of the placing of a palatal obturator and the suggestion of some oral-hygiene habits [24, 25]. A palatal obturator is a device that possesses the following several therapeutic effects: (1) it enhances the esthetic result of nasolabial structures and reduces the need for additional surgical procedures by bringing the soft and bone structures surrounding the palatal cleft closer together [25–27]; (2) it creates a seal between the oral and nasal cavities to control the flow of liquids and solid foods [28]; and (3) it restores some basic oral functions such as chewing, swallowing, and speech [25, 28]. Additionally, the obturator creates a rigid surface that allows the child to press her/his mother's nipple and create sufficient negative pressure to achieve proper suction of breast milk, facilitating the feeding process [24, 28]. The device also reduces nasal regurgitation and the possibility of asphyxiation and aids in achieving correct positioning of the tongue, thus enhancing the functional development of the maxillaries and speech [26]. Because of the horizontal position of the Eustachian tube, abnormal insertion of elevators and soft palate tensor, and the child's muscle hypoplasia, all characteristic findings of patients with ABS, a permanent significant risk is present of food passing through the nasopharynx [25]. The palatal obturator reduces such a risk, thus the incidence of otitis media and nasopharynx infections [24]. These orthopedic devices are considered essential during the presurgical phase, a technique initially developed by McNeil and Burston [24, 26]. Due to all of these preventive and therapeutic reasons, we placed the palatal obturator in our patient prior to the reconstructive procedures.

It is also noteworthy that the oral cavity of an edentate child, such as the patient mentioned herein, should be cleaned at least once a day, preferably at night, using wet gauze with saline solution or filtered water on the alveolar ridges [20]. If the mouth is cleaned each day, the child will grow up with the sensation of having a healthy mouth and will become accustomed to the manipulation of the oral cavity's soft and hard structures [22]. Once the primary teeth have erupted, a special toothbrush with soft bristles for infants should be used, and later, an electrical toothbrush may be implemented; this recommendation would be especially useful in cases of children with motor or neuronal alterations [12].

## 5. Conclusions

Pediatric Dentists have the obligation of possessing the essential knowledge of the ABS, not only to advise the patient's parents, but also to refer the child to other health professionals. Additionally, active participation by the practitioner is necessary in the management process of ABS patients, for instance, timely diagnosis, prevention, and treatment of the different craniofacial/oral anomalies, control of the growth and development of teeth and maxillary area, and collaboration during the correction of the diverse structural anomalies. In order to achieve these objectives, it is essential to obtain an exhaustive medical history, which allows the specialist to evaluate thoroughly all craniofacial anomalies, design a proper dental treatment plan, and prevent possible complications during the management of these vulnerable patients.

## References

[1] Hotwani K., Sharma K. (2015). Oral rehabilitation for amniotic band syndrome: an unusual presentation. *International Journal of Clinical Pediatric Dentistry*.

[2] Koskimies E., Syvänen J., Nietosvaara Y., Mäkitie O., Pakkasjärvi N. (2015). Congenital constriction band syndrome with limb defects. *Journal of Pediatric Orthopaedics*.

[3] Eppley B. L., David L., Li M., Moore C. A., Sadove A. M. (1998). Amniotic band facies. *The Journal of Craniofacial Surgery*.

[4] Doi Y., Kawamata H., Asano K., Imai Y. (2011). A case of amniotic band syndrome with cleft lip and palate. *Journal of Maxillofacial and Oral Surgery*.

[5] Streeter G. L. (1930). *Carnegie Institution Focal deficiencies in fetal tissues and their relation to intra-uterine amputation of Washington*.

[6] Torpin R. (1968). *Fetal Malformations caused by Amnion Rupture during Gestation*.

[7] Kino Y. (1975). Clinical and experimental studies of the congenital constriction band syndrome, with an emphasis on its etiology. *Journal of Bone and Joint Surgery*.

[8] Coady M. S. E., Moore M. H., Wallis K. (1998). Amniotic band syndrome: the association between rare facial clefts and limb ring constrictions. *Plastic and Reconstructive Surgery*.

[9] Purandare S. M., Ernst L., Medne L., Huff D., Zackai E. H. (2009). Developmental anomalies with features of disorganization (Ds) and amniotic band sequence (ABS): a report of four cases. *American Journal of Medical Genetics, Part A*.

[10] Muraskas J. K., McDonnell J. F., Chudik R. J., Salyer K. E., Glynn L. (2003). Amniotic band syndrome with significant orofacial clefts and disruptions and distortions of craniofacial structures. *Journal of Pediatric Surgery*.

[11] Bouguila J., Ben Khoud N., Ghrissi A. (2007). Amniotic band syndrome and facial malformations. *Revue de Stomatologie et de Chirurgie Maxillo-Faciale*.

[12] Coyle S., Karp J. M., Shirakura A. (2008). Oral rehabilitation of a child with amniotic band syndrome. *Journal of Dentistry for Children*.

[13] Das D., Das G., Gayen S., Konar A. (2011). Median facial cleft in amniotic band syndrome. *Middle East African Journal of Ophthalmology*.

[14] Hukki J., Balan P., Ceponiene R., Kantola-Sorsa E., Saarinen P., Wikstrom H. (2004). A case study of amnion rupture sequence with acalvaria, blindness, and clefting: clinical and psychological profiles. *The Journal of Craniofacial Surgery*.

[15] Pedersen T. K., Thomsen S. G. (2001). Spontaneous resolution of amniotic bands. *Ultrasound in Obstetrics and Gynecology*.

[16] Morovic C. G., Berwart F., Varas J. (2004). Craniofacial anomalies of the amniotic band syndrome in serial clinical cases. *Plastic and Reconstructive Surgery*.

[17] Buccoliero A. M., Castiglione F., Garbini F. (2011). Amniotic band syndrome: a case report. *Pathologica*.

[18] Cignini P., Giorlandino C., Padula F., Dugo N., Cafà E. V., Spata A. (2012). Epidemiology and risk factors of amniotic band syndrome, or ADAM sequence. *Journal of Prenatal Medicine*.

[19] Obdeijn M. C., Offringa P. J., Bos R. R. M., Verhagen A. A. E., Niessen F. B., Roche N. A. (2010). Facial clefts and associated limb anomalies: description of three cases and a review of the literature. *Cleft Palate-Craniofacial Journal*.

[20] Taub P. J., Bradley J. P., Setoguchi Y., Schimmenti L., Kawamoto H. K. (2003). Typical facial clefting and constriction band anomalies: an unusual association in three unrelated patients. *American Journal of Medical Genetics*.

[21] Perlyn C. A., Schmelzer R., Govier D., Marsh J. L. (2005). Congenital scalp and calvarial deficiencies: principles for classification and surgical management. *Plastic and Reconstructive Surgery*.

[22] Jabor M. A., Cronin E. D. (2000). Bilateral cleft lip and palate and limb deformities: a presentation of amniotic band sequence?. *Journal of Craniofacial Surgery*.

[23] Adeosun O. O., James D., Akinmoladun V. I., Owobu T. (2012). Amniotic band syndrome associated with orofacial clefts: a report of two cases. *Oral Surgery*.

[24] Narendra R., Sashi Purna C. R., Reddy S. D., Simhachalam Reddy N., Sesha Reddy P., Rajendra Prasad B. (2013). Feeding obturator-a presurgical prosthetic aid for infants with cleft lip and palate-clinical report. *Annals and Essences of Dentistry*.

[25] Goyal M., Chopra R., Bansal K., Marwaha M. (2014). Role of obturators and other feeding interventions in patients with cleft lip and palate: a review. *European Archives of Paediatric Dentistry*.

[26] Ravichandra K. S., Vijayaprasad K. E., Vasa A. A. K., Suzan S. (2010). A new technique of impression making for an obturator in cleft lip and palate patient. *Journal of Indian Society of Pedodontics and Preventive Dentistry*.

[27] Papadopoulos M. A., Koumpridou E. N., Vakalis M. L., Papageorgiou S. N. (2012). Effectiveness of pre-surgical infant orthopedic treatment for cleft lip and palate patients: a systematic review and meta-analysis. *Orthodontics & Craniofacial Research*.

[28] Britton K. F. M., McDonald S. H., Welbury R. R. (2011). An investigation into infant feeding in children born with a cleft lip and/or palate in the West of Scotland. *European Archives of Paediatric Dentistry*.

